# Utilization of the Abbott SARS-CoV-2 IgG II Quant Assay To Identify High-Titer Anti-SARS-CoV-2 Neutralizing Plasma against Wild-Type and Variant SARS-CoV-2 Viruses

**DOI:** 10.1128/spectrum.02811-22

**Published:** 2022-09-20

**Authors:** Yi-Chan J. Lin, David H. Evans, Ninette F. Robbins, Guillermo Orjuela, Queenie Hu, Reuben Samson, Kento T. Abe, Bhavisha Rathod, Karen Colwill, Anne-Claude Gingras, Ashleigh Tuite, Qi-Long Yi, Sheila F. O’Brien, Steven J. Drews

**Affiliations:** a Department of Medical Microbiology & Immunology, University of Alberta, Edmonton, Alberta, Canada; b Scientific Affairs, Abbott Transfusion Medicine, Chicago, Illinois, USA; c Scientific Affairs, Abbott Transfusion Medicine, Bogotá, Colombia; d Lunenfeld-Tanenbaum Research Institute at Mt. Sinai Hospital, Sinai Health, Toronto, Ontario, Canada; e Department of Molecular Genetics, University of Toronto, Toronto, Ontario, Canada; f Dalla Lana School of Public Health, University of Toronto, Toronto, Ontario, Canada; g Epidemiology and Surveillance, Canadian Blood Services, Ottawa, Ontario, Canada; h School of Epidemiology and Public Health, University of Ottawa, Ottawa, Ontario, Canada; i Canadian Blood Services, Microbiology, Edmonton, Alberta, Canada; j Department of Laboratory Medicine and Pathology, University of Alberta, Edmonton, Alberta, Canada; Johns Hopkins Hospital

**Keywords:** COVID-19 convalescent plasma, SARS-CoV-2 antibody, neutralizing antibody, plaque reduction neutralization, method comparisons

## Abstract

There is evidence that COVID-19 convalescent plasma may improve outcomes of patients with impaired immune systems; however, more clinical trials are required. Although we have previously used a 50% plaque reduction/neutralization titer (PRNT_50_) assay to qualify convalescent plasma for clinical trials and virus-like particle (VLP) assays to validate PRNT_50_ methodologies, these approaches are time-consuming and expensive. Here, we characterized the ability of the Abbott severe acute respiratory syndrome coronavirus 2 (SARS-CoV-2) IgG II Quant assay to identify high- and low-titer plasma for wild-type and variant (Alpha, Beta, Gamma, and Delta) SARS-CoV-2 characterized by both VLP assays and PRNT_50_. Plasma specimens previously tested in wild-type, Alpha, Beta, Gamma, and Delta VLP neutralization assays were selected based on availability. Selected specimens were evaluated by the Abbott SARS-CoV-2 IgG II Quant assay [Abbott anti-Spike (S); Abbott, Chicago, IL], and values in units per milliliter were converted to binding antibody units (BAU) per milliliter. Sixty-three specimens were available for analysis. Abbott SARS-CoV-2 IgG II Quant assay values in BAU per milliliter were significantly different between high- and low-titer specimens for wild-type (Mann-Whitney U = 42, *P* < 0.0001), Alpha (Mann-Whitney U = 38, *P* < 0.0001), Beta (Mann-Whitney U = 29, *P* < 0.0001), Gamma (Mann-Whitney U = 0, *P* < 0.0001), and Delta (Mann-Whitney U = 42, *P* < 0.0001). A conservative approach using the highest 95% confidence interval (CI) values from wild-type and variant of concern (VOC) SARS-CoV-2 experiments would identify a potential Abbott SARS-CoV-2 IgG II Quant assay cutoff of ≥7.1 × 10^3^ BAU/mL.

**IMPORTANCE** The United States Food and Drug Administration (FDA) issued an Emergency Use Authorization (EUA) for the use of COVID-19 convalescent plasma (CCP) to treat hospitalized patients with COVID-19 in August 2020. However, by 4 February 2021, the FDA had revised the convalescent plasma EUA. This revision limited the authorization for high-titer COVID-19 convalescent plasma and restricted patient groups to hospitalized patients with COVID-19 early in their disease course or hospitalized patients with impaired humoral immunity. Traditionally our group utilized 50% plaque reduction/neutralization titer (PRNT_50_) assays to qualify CCP in Canada. Since that time, the Abbott SARS-CoV-2 IgG II Quant assay (Abbott, Chicago IL) was developed for the qualitative and quantitative determination of IgG against the SARS-CoV-2. Here, we characterized the ability of the Abbott SARS-CoV-2 IgG II Quant assay to identify high- and low-titer plasma for wild-type and variant (Alpha, Beta, Gamma, and Delta) SARS-CoV-2.

## INTRODUCTION

The United States Food and Drug Administration (FDA) issued an Emergency Use Authorization (EUA) for the use of COVID-19 convalescent plasma (CCP) to treat hospitalized patients with COVID-19 in August 2020. However, by 4 February 2021, the FDA had revised the convalescent plasma EUA. This revision limited the authorization for high-titer CCP to hospitalized patients with COVID-19 early in disease or hospitalized patients with impaired humoral immunity. There were also recommendations against the use of CCP for treating COVID-19 in “hospitalized patients without impaired humoral immunity” (1). Our group at Canadian Blood Services previously was focused on supporting CCP studies in Canada (2–5), and this led us to initiate a “correlates of immunity” project, which had the stated goal of understanding changes in anti-severe acute respiratory syndrome coronavirus 2 (anti-SARS-CoV-2) neutralizing capacity as the COVD-19 pandemic advanced and identifying methods to identify and characterize high-titer donor plasma (2, 3, 6, 7). During this preliminary work, we validated 50% plaque reduction/neutralization titer (PRNT_50_) assays using binding antibody, competitive binding assays, and virus-like particle (VLP) assays for wild-type SARS-CoV-2 (3).

Our group qualified CCP to Canada-based clinical trials initially using a PRNT_50_ of ≥1:160 with wild-type SARS-CoV-2 (2, 4, 5), which prior to vaccine rollout was a substantial titer (2). At first, results of CCP studies partly supported by our group were discouraging. The Randomized, Embedded, Multifactorial, Adaptive Platform Trial for Community-Acquired Pneumonia (REMAP-CAP) was unable to show an association between administration of ABO-compatible CCP and an improvement in the number of organ support-free days in critically ill patients (4). In hospitalized patients with COVID-19, the Convalescent Plasma for COVID-19 Respiratory Illness (CONCOR-1) trial determined that CCP was not associated with a reduced risk of intubation or death (at 30 days) (5). Given the diversity of the study sites, CCP was qualified using PRNT_50_ by Canadian Blood Services but also by enzyme-linked immunosorbent assay (ELISA) assays by other blood operators. That clinical trial found that transfusion of convalescent plasma with unfavorable antibody profiles, including low anti-SARS-CoV-2 neutralizing capacity or low levels of antibody-dependent cellular cytotoxicity (ADCC), might be associated with worse clinical outcomes compared to standard care (5). Recently, the COMPILE study pooled individual patient data from eight international randomized control trials (8). The COMPILE study determined that patients at an early COVID-19 stage, including those with a preexisting condition (e.g., diabetes and/or cardiovascular or pulmonary disease) were expected to benefit most from CCP (9). In another study, it was shown that the provision of CCP to unvaccinated individuals early in infection may also reduce the risk of hospitalization (10).

Our experience qualifying CCP provided some lessons on laboratory workflow and efficiency. We found that prior to SARS-CoV-2 vaccine rollout, many postinfection CCP donors were not able to produce high-titer CCP or that donations over time (e.g., 3 to 4 months from initial donation) would drop in titer and become ineligible for the clinical trials (2). Given the labor-intensive nature of PRNT_50_ testing, we also noted a requirement for a laboratory-based rapid screening assay to identify high-titer CCP and to decrease overall tests costs per unit of qualified CCP. We also later determined that the neutralizing capacity of donor plasma could vary between wild-type and emerging variants of concern (VOCs) (7). The rationale to use CCP in early stage COVID-19 is further supported in the study that SARS-CoV-2 virus can only be cultured from early stage COVID-19 patients (11).

The Abbott SARS-CoV-2 IgG II Quant assay [Abbott anti-Spike (S), Abbott, Chicago, IL, USA; here referred to as the Abbott Quant assay] was developed for the qualitative and quantitative determination of IgG against the SARS-CoV-2 S (12). This assay has been used in multiple seroprevalence surveys (13–16). Here, we characterized the ability of the Abbott Quant assay to identify high- and low-titer plasma for wild-type and variant (Alpha, Beta, Gamma, and Delta) SARS-CoV-2, as analyzed by a virus-like particle assay and PRNT_50_.

## RESULTS

### Study population characteristics.

As previously described, study specimens were sampled from a larger repeated cross-sectional design with random cross-sectional sampling of all available retention samples (*n* = 1,500/month) and previously tested for neutralization of wild-type and VOC VLPs (7) (see the supplemental material). Summaries of prior wild-type immunoassay results used to select specimens for further analyses are listed in Table S1 in the supplemental material. A summary of wild-type and VOC 50% infective dose (ID_50_) results (January to March 2021) previously generated (7) in vaccinated and unvaccinated blood donors for January to March 2021 is listed in Table S2.

From the specimens analyzed in the prior VLP neutralization study, 63 were available for the current analysis by PRNT_50_ (using wild-type, Alpha, Beta, Gamma, and Delta SARS-CoV-2) and the Abbott Quant assay. These 63 specimens, their associated donor vaccination histories, as well as the specimen anti-nucleocapsid protein antibody (anti-N) profiles are presented in Tables S3 to S6. Infections were likely wild type or Alpha based on the timing of specimen collection (January to March 2021) (17). Importantly, these samples are from a random sampling of the blood donor population instead of a specific selection for convalescent blood therapy donations.

### SARS-CoV-2-vaccinated blood donors.

As per Tables S3 and S4, there were 25 donors with a self-declared history of SARS-CoV-2 vaccination. Seventeen (68.0%) had no further information on dosing or timing. Six (24.0%) had stated that they had received one dose ≥14 days prior to donation. Two donors (8.0%) indicated that they were “fully vaccinated,” which might have been indicative of two doses at the time but cannot be verified. Given the small numbers of fully vaccinated individuals and to focus on the use of the Abbott Quant assay as a surrogate neutralization assay, this study did not attempt to compare the differences in PRNT_50_ neutralization of wild-type, Alpha, Beta, Gamma, and Delta SARS-CoV-2 within or between vaccinated and unvaccinated groups.

### Correlations of Abbott Quant assay log BAU per milliliter versus log PRNT_50_.

As PRNT_50_ results were not considered continuous data, nonparametric correlations between Abbott Quant assay log binding antibody unit (BAU)-per-milliliter values and log PRNT_50_ values (using wild-type, Alpha, Beta, Gamma, and Delta SARS-CoV-2) were determined. In this series of experiments, the highest correlations were found between the log BAU-per-milliliter values determined by the Abbott Quant assay and wild-type log PRNT_50_ values (Spearman *r* = 0.89; 95% confidence interval [CI], 0.818 to 0.93; 55 pairs; *P* < 0.0001). This was then followed by Alpha log PRNT_50_ versus Alpha VLP log ID_50_ (Spearman *r* = 0.85; 95% CI, 0.75 to 0.91; 55 pairs; *P* < 0.0001), Delta log PRNT_50_ versus Delta VLP log ID_50_ (Spearman *r* = 0.79; 95% CI, 0.66 to 0.88, 55 pairs; *P* < 0.0001), Beta log PRNT_50_ versus Beta VLP log ID_50_ (Spearman *r* = 0.78; 95% CI, 0.65 to 0.87; 55 pairs; *P* < 0.0001), and Gamma log PRNT_50_ versus Gamma VLP log ID_50_ (Spearman *r* = 0.70; 95% CI, 0.52 to 0.82; 55 pairs; *P* < 0.0001).

### Correlations of Abbott Quant assay log BAU per milliliter versus VLP log ID_50_.

To ensure that correlations between the Abbott SARS-CoV-2 IgG II Quant assay and SARS-CoV-2 neutralization assays results were generalizable, correlations between the Abbott SARS-CoV-2 IgG II Quant assay and VLP assay results were also assessed for wild-type and VOC SARS-CoV-2. In this series of experiments, the ordinal ranking of correlations matched the Abbott Quant assay and VLP analyses. The highest correlations were found between the BAU-per-milliliter values determined by the Abbott Quant assay and wild-type VLP log ID_50_ values (Spearman *r* = 0.92; 95% CI, 0.86 to 0.95; 55 pairs; *P* < 0.0001). This was then followed by Abbott Quant assay versus Alpha VLP log ID_50_ (Spearman *r* = 0.87; 95% CI, 0.78 to 0.92; 55 pairs; *P* < 0.0001), Abbott Quant assay versus Delta VLP log ID_50_ (Spearman *r* = 0.85; 95% CI, 0.75 to 0.91; 55 pairs; *P* < 0.0001), Abbott Quant assay versus Beta VLP log ID_50_ (Spearman *r* = 0.75; 95% CI, 0.60 to 0.84; 55 pairs; *P* < 0.0001) and Abbott Quant assay versus Gamma VLP log ID_50_ (Spearman *r* = 0.75; 95% CI, 0.60 to 0.85;, 55 pairs; *P* < 0.0001).

### Correlation of PRNT_50_ and VLP results for wild-type, Alpha, Beta, Gamma, and Delta SARS-CoV-2.

To further assess the generalizability of the neutralization assay results, correlations between PRNT_50_ and VLP results were assessed. PRNT_50_ and VLP results for wild-type and VOC SARS-CoV-2 were strongly correlated and were as follows: wild type, Spearman *r* = 0.96, 95% CI, 0.93 to 0.97, 63 pairs, *P* < 0.0001; Alpha, Spearman *r* = 0.95, 95% CI, 0.92 to 0.97, 63 pairs, *P* < 0.0001; Beta, Spearman *r* = 0.83, 95% CI, 0.74 to 0.90, 63 pairs, *P* < 0.0001; Gamma, Spearman *r* = 0.84, 95% CI, 0.74 to 0.90, 63 pairs, *P* < 0.0001; and Delta, Spearman *r* = 0.91, 95% CI, 0.85 to 0.94, 63 pairs, *P* < 0.0001.

### Overlaying PRNT_50_ results on Abbott Quant assay BAU-per-milliliter versus wild-type, Alpha, Beta, Gamma, and Delta VLP log ID_50_ results.

**FIG 1:**
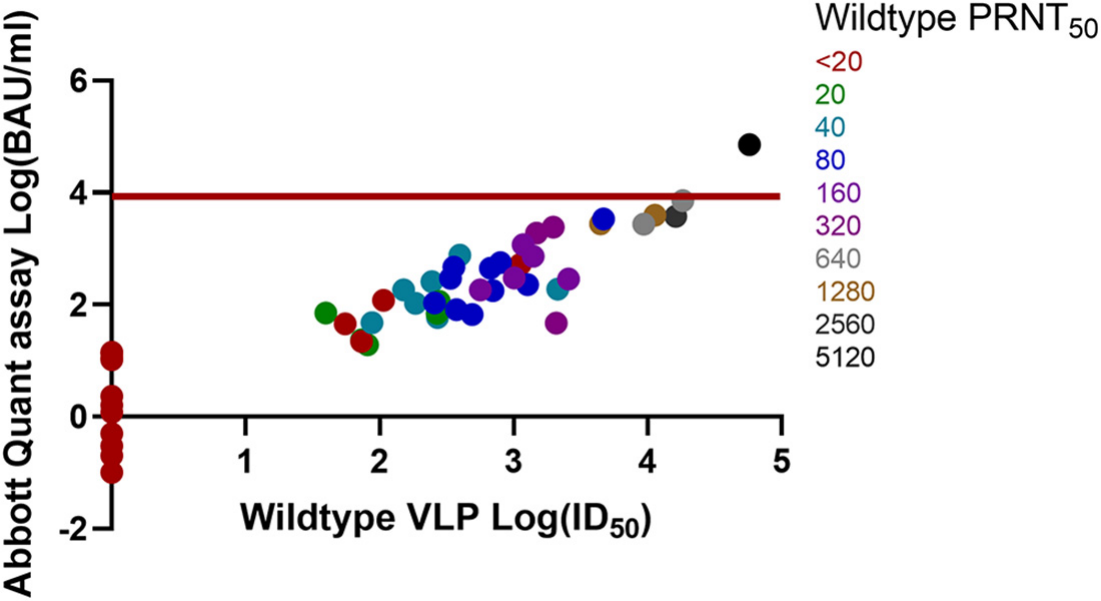
Comparison of Abbott Quant assay, wild-type SARS-CoV-2 VLP, and wild-type PRNT_50_ methodologies on donor plasma specimens. This graph compares Abbott Quant assay log BAU-per-milliliter results on the *y* axis against wild-type SARS-CoV-2 VLP assay log ID_50_ results on the *x* axis. PRNT_50_ results are overlaid on specimen-specific data points and color coded for wild-type SARS-CoV-2 PRNT_50_ titers. Fifty-five pairs of data were quantifiable after log transformation of both BAU-per-milliliter and wild-type ID_50_ results. The red horizontal line identifies a potential Abbott Quant assay cutoff of ≥7.1 × 10^3^ BAU/mL or log_3.9_ BAU/mL.

**FIG 2:**
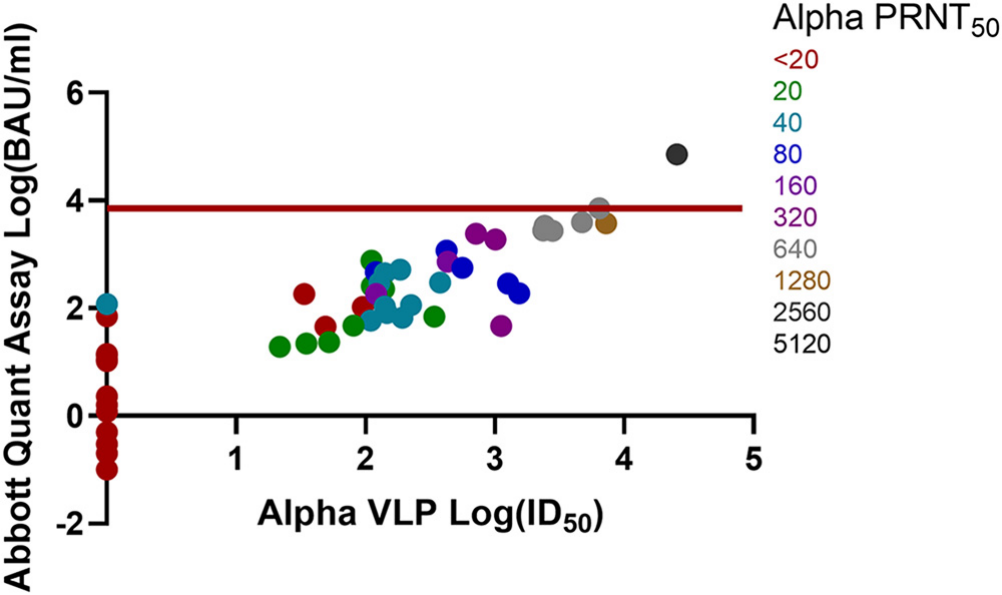
Comparison of Abbott Quant assay, Alpha SARS-CoV-2 VLP, and Alpha PRNT_50_ methodologies on donor plasma specimens. This graph compares Abbott Quant assay log BAU-per-milliliter results on the *y* axis against Alpha SARS-CoV-2 VLP assay log ID_50_ results on the *x* axis. PRNT_50_ results are overlaid on specimen-specific data points and color coded for Alpha SARS-CoV-2 PRNT_50_ titers. Fifty-five pairs of data were available after log transformation of both BAU-per-milliliter and Alpha ID_50_ results. The red horizontal line identifies a potential Abbott Quant assay cutoff of ≥7.1 × 10^3^ BAU/mL or log_3.9_ BAU/mL.

**FIG 3:**
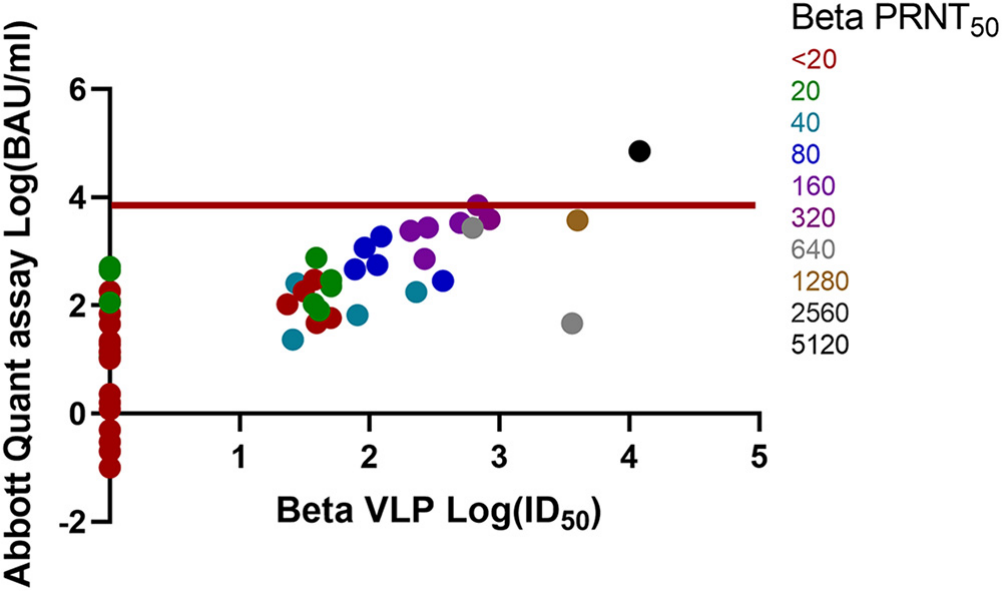
Comparison of Abbott Quant assay, Beta SARS-CoV-2 VLP, and Beta PRNT_50_ methodologies on donor plasma specimens. This graph compares Abbott Quant assay log BAU-per-milliliter results on the *y* axis against Beta SARS-CoV-2 VLP assay log ID_50_ results on the *x* axis. PRNT_50_ results are overlaid on specimen-specific data points and color coded for Beta SARS-CoV-2 PRNT_50_ titers. Fifty-five pairs of data were available after log transformation of both BAU-per-milliliter and Beta ID_50_ results. The red horizontal line identifies a potential Abbott Quant assay cutoff of ≥7.1 × 10^3^ BAU/mL or log_3.9_ BAU/mL.

**FIG 4:**
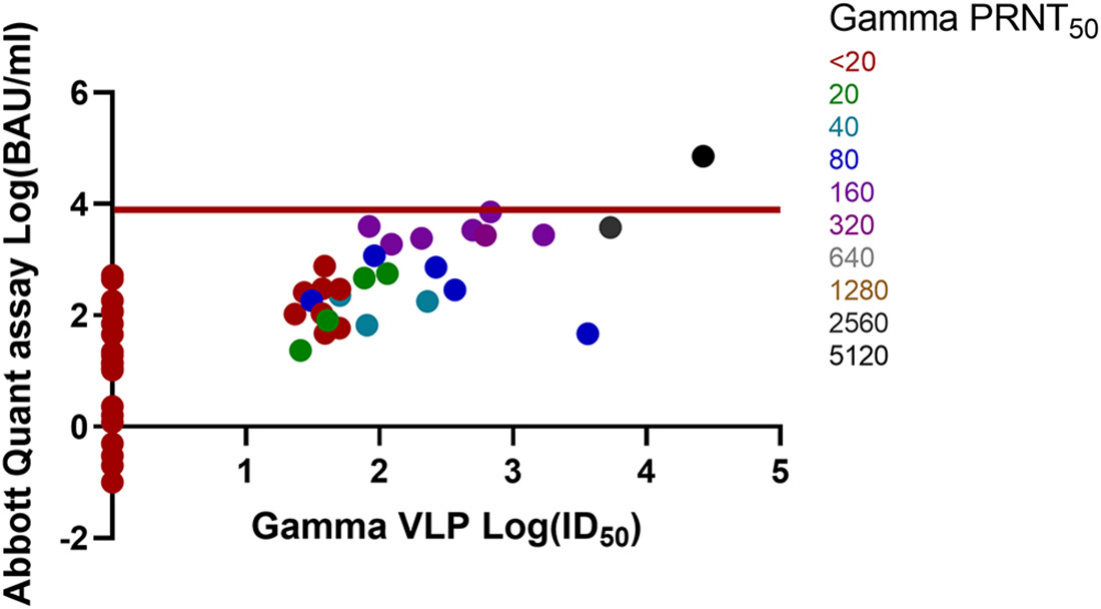
Comparison of Abbott Quant assay, Gamma SARS-CoV-2 VLP, and Gamma PRNT_50_ methodologies on donor plasma specimens. This graph compares Abbott Quant assay log BAU-per-milliliter results on the *y* axis against Gamma SARS-CoV-2 VLP assay log ID_50_ results on the *x* axis. PRNT_50_ results are overlaid on specimen-specific data points and color coded for Gamma SARS-CoV-2 PRNT_50_ titers. Fifty-five pairs of data were available after log transformation of both BAU-per-milliliter and Gamma ID_50_ results. The red horizontal line identifies a potential Abbott Quant assay cutoff of ≥7.1 × 10^3^ BAU/mL or log_3.9_ BAU/mL.

**FIG 5:**
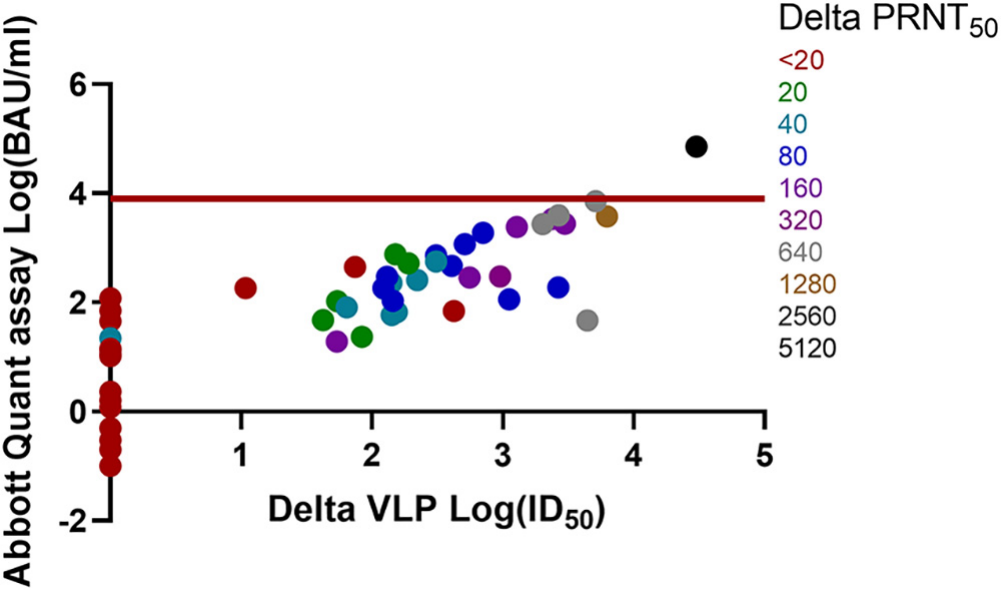
Comparison of Abbott Quant assay, Delta SARS-CoV-2 VLP, and Delta PRNT_50_ methodologies on donor plasma specimens. This graph compares Abbott Quant assay log BAU-per-milliliter results on the *y* axis against Delta SARS-CoV-2 VLP assay log ID_50_ results on the *x* axis. PRNT_50_ results are overlaid on specimen-specific data points and color coded for Delta SARS-CoV-2 PRNT_50_ titers. Fifty-five pairs of data were available after log transformation of both BAU-per-milliliter and Delta ID_50_ results. The red horizontal line identifies a potential Abbott Quant assay cutoff of ≥7.1 × 10^3^ BAU/mL or log_3.9_ BAU/mL.

Comparisons of the Abbott Quant assay BAU-per-milliliter versus wild-type, Alpha, Beta, Gamma, and Delta VLP log ID_50_ and PRNT_50_ results are shown in Fig. 1, Fig. 2, Fig. 3, Fig. 4, and Fig. 5. Abbott Quant assay results are presented on the *y* axes, and VLP log_50_ results are presented on *x* axes. As PRNT_50_ results were not considered continuous data, color-coded PRNT_50_ values for each specimen are overlaid on corresponding data points. In general, specimens that had high-titer PRNT_50_ results clustered to the upper-right-hand side of the graph as expected. In contrast, specimens with lower PRNT_50_ results clustered to the lower-left-hand side of the graph.

### Difference in Abbott Quant assay BAU-per-milliliter values between high- and low-titer plasma as defined by wild-type and VOC PRNT_50_ assays.

Abbott Quant assay BAU-per-milliliter values were significantly different between high-titer (PRNT_50_, ≥1:160) and low-titer (PRNT_50_, <1:160) specimens for the wild type (Mann-Whitney U = 42, *P* < 0.0001), Alpha (Mann-Whitney U = 38, *P* < 0.0001), Beta (Mann-Whitney U = 29, *P* < 0.0001), Gamma (Mann-Whitney U = 0, *P* < 0.0001), and Delta (Mann-Whitney U = 42, *P* < 0.0001). All tests were two tailed.

### Identification of high-titer plasma using the Abbott Quant assay.

In Table 1, we identified the median and 95% CI lower and upper ranges for donor plasma with wild-type and VOC PRNT_50_ values of ≥160. Use of an Abbott Quant assay result of ≥3,392.0 BAU/mL would be representative of the highest median neutralization value, corresponding to Gamma SARS-CoV-2 in the group of wild-type and VOC SARS-CoV-2 assessed against Gamma SARS-CoV-2. A more conservative approach at identifying the highest 95% CI value from wild-type and VOC SARS-CoV-2 experiments would identify a potential Abbott SARS-CoV-2 IgG II Quant assay cutoff of ≥7.1 × 10^3^ BAU/mL or log_3.9_ BAU/mL expressed in Fig. 1 to 5.

**TABLE 1:** Characterization of high- and low-titer plasma using the Abbott Quant assay

Strain	Result at PRNT_50_ of:					
	<160			≥160		
	Anti-S protein median BAU/mL	95% CI	*N*	Anti-S protein median BAU/mL	95% CI	*N*
Wild type	20.8	1.2–70.7	48	2,432	301.8–3,790	15
Alpha	23.6	2.3–81.9	51	2,762	728.7–3,936	12
Beta	45.7	2.3–106.8	53	3,088	728.7–7,143	10
Gamma	46.2	10.4–105.7	54	3,392	2,432–7,143	9
Delta	34.7	2.3–105.7	52	2,783	285.7–7,143	11

## DISCUSSION

Here, we describe the test characteristics of the Abbott SARS-CoV-2 IgG II Quant assay against VLP and PRNT_50_ methodologies for neutralization of wild-type, Alpha, Beta, Gamma, and Delta variants. This chemiluminescent microparticle immunoassay for IgG against the receptor binding domain (RBD) region of S has an analytical measuring interval of 21.0 to 40,000 AU/mL (3 to 5,714 BAU/mL), an extended measuring interval of 40,000 to 80,000 AU/mL (5,714 to 1,1429 BAU/mL), and a reportable interval of 6.8 to 80,000 AU/mL (1 to 11,429 BAU/mL) (12, 18). We also identified Abbott SARS-CoV-2 IgG II Quant assay cutoffs that could be used to qualify high-titer plasma against these variants. This work is important because it allows us to consider the use of the SARS-CoV-2 IgG II Quant assay to qualify plasma CCP products for use in clinical trials involving patients with a clinical preexisting condition (e.g., diabetes and/or cardiovascular or pulmonary disease) (9).

Our study also was undertaken during a period when seroprevalence to SARS-CoV-2 was extremely low and when most Canadians with a history of SARS-CoV-2 infection would have been infected with wild-type SARS-CoV-2 (17). Prior to SARS-CoV-2 vaccine rollout, in November 2020, both latent class analysis and a composite reference standard analysis identified a SARS-CoV-2 seroprevalence of <2.5% among Canadian blood donors (19). The majority (68%) of donors with a self-declared history of SARS-CoV-2 vaccination provided no information on dosing or timing. Another 24.0% had stated that they had received one dose ≥14 days prior to donation. Only a minority (8%) of donors claimed that they were “fully vaccinated,” but no other information was provided. At that time period, a fully vaccinated Canadian donor would have received two doses of a Health Canada-approved vaccine (20). As of 27 March 2021, 10.11% of the Canadian population had received one dose of vaccine and 1.75% were fully vaccinated (20). Here, the focus was on characterizing the SARS-CoV-2 IgG II Quant assay as a surrogate quantitative assay while still providing context to the origins of the specimens.

All available donor specimens were tested with the Abbott SARS-CoV-2 IgG II Quant assay and wild-type and VOC PRNT_50_ assays and then compared to prior wild-type and VOC VLP results. Because VLP assays generated continuous data and not doubling dilutions, we assessed the correlations between the BAU per milliliter generated by the Abbott Quant assay and IC_50_ values generated in VLP assays (3). In this series of experiments, the rank-ordered correlations between the Abbott Quant assay and both neutralization assays were wild type > Alpha > Delta > Beta > Gamma. This ordinal drop in correlations might suggest that the Abbott Quant assay has some bias for quantifying wild-type S antigen and reflects the molecular evolution of the S protein (21). This would suggest that qualification of donor plasma for CCP should consider the SARS-CoV-2 strain to be neutralized.

Prior to this study, we saw a strong relationship between PRNT_50_ results and VLP results for wild-type SARS-CoV-2 (3). We then used PRNT_50_ results to qualify convalescent plasma for clinical trials (2, 4, 5, 22). The work in this article suggests that PRNT_50_ results were also generalizable to other neutralization approaches (e.g., VLP assays) for wild-type and VOC SARS-CoV-2. Strong correlations between the Abbott Quant assay, VLP, and PRNT_50_ assays regardless of the SARS-CoV-2 strain neutralized (e.g., wild-type, Alpha, Beta, Gamma, and Delta SARS-CoV-2) suggest that a general CCP cutoff could be estimated for the Abbott SARS-CoV-2 IgG II Quant assay using a cutoff correction. This is further supported by a visual assessment suggesting an upper-right-hand distribution of PRNT_50_ higher-titer specimens in Fig. 1 to 5.

A general cutoff for the Abbott SARS-CoV-2 IgG II Quant assay could be estimated by comparing median and upper 95% CI BAU-per-milliliter results against PRNT_50_ results of ≥1:160 against wild-type and VOC SARS-CoV-2. In Table 1, we identified median and 95% CI lower and upper ranges for donor plasma with wild-type and VOC PRNT_50_ values of ≥160. Use of an Abbott SARS-CoV-2 IgG II Quant assay result of ≥3,392.0 BAU/mL would be representative of the highest median neutralization value, corresponding to Gamma SARS-CoV-2 in the group of wild-type and VOC SARS-CoV-2 assessed. A more conservative approach to identifying the highest 95% CI value from wild-type and VOC SARS-CoV-2 experiments would identify a potential Abbott SARS-CoV-2 IgG II Quant assay cutoff of ≥7.1 × 10^3^ BAU/mL (or log_3.9_ BAU/mL) expressed in Fig. 1 to 5. As of May 2022, these cutoff values should be easily achievable in the blood donor population. A recent Canadian Blood Services seroprevalence analysis (1 to 15 May 2022) determined that median titers in Canadians aged 17 to 70+ years (*n* = 15,958 blood donors) ranged around 10^4^ BAU/mL using a different test platform (23).

There are a variety of neutralization assays that have been described to be able to measure functional antibody neutralization against wild-type as well as variant SARS-CoV-2 that involve binding proxy assays (24–27), pseudovirus, or VLP neutralization (7, 25, 26) as well as SARS-CoV-2 culture-based neutralization (26, 28). Not all methods compare wild-type or variant SARS-CoV-2 neutralization. Temporal and population context (here blood donors in the early part of 2021) are also important. Comparisons between studies will also be affected by whether populations were analyzed after natural infection, vaccination, or a mix of both infection and vaccination (29). This variability described above means that comparisons to work by other groups may at first appear to be complicated. Although immunoassays vary by target measured (e.g., IgG, IgM, and IgA), many can be cross compared using a conversion of individual assay results to BAU per milliliter (18). A prior group (Benites et al.) used the Abbott IgG assay to identify a threshold of 137.65 AU/mL (20 BAU/mL) for detecting neutralizing antibodies at ≥1:80 against wild-type SARS-CoV-2 in a cell culture-based neutralization assay (30). Benites et al., also utilized a small number of specimens but chose a lower neutralization dilution (≥1:80) and did not assess neutralization of variant SARS-CoV-2 (30). Another group (Priddy et al.) found that titers of 8,558 to 9,514 AU/mL (1,223 to 1,359 BAU/mL) of the Abbott IgG assay corresponded to 94.5% neutralization using a surrogate competitive neutralization assay (Genscript, Singapore) against wild-type RBD (31). This would be within a similar range to our estimated neutralization of wild-type SARS-CoV-2. The work by Harvala et al. is probably most relevant to our study and suggests when using the Roche anti-S assay (which measures total antibody versus RBD/Spike) that a value of 20,000 BAU/mL corresponded to a neutralization of Delta at a concentration deemed relevant to CCP studies (≥1:640 using a different neutralization protocol) (28).

There are some important caveats to this study. We used a relatively small number of specimens for the time January to March 2021 (7). Donor-declared histories of vaccination were often incomplete, and therefore, we were unable to extensively characterize the ability of the Abbott SARS-CoV-2 IgG II Quant assay (BAU per milliliter) to make a distinction between individuals with natural infection, vaccination, and a combination of both natural infection and vaccination. This was an issue as we did not have access to the health and vaccination databases where our donors lived. This work began during a period when Delta was the predominant SARS-CoV-2 VOC. The work described here does not describe a potential threshold for the Abbott Quant assay to detect high neutralizing capacity against Omicron SARS-CoV-2, a diverse group of variants in which antibody neutralization is also expected to be variable. This was due to the time lag required to undertake PRNT_50_ for newly emerging Omicron VOCs as well as the need to utilize a different cell culture approach for undertaking Omicron PRNT_50_ assays. At the time that we performed this study, we did not have access to Omicron-specific VLPs. Note, however, that we and other groups have since demonstrated a further decrease in neutralization of Omicron in comparison to the VLPs used in the current study (32–34). Also, growth characteristics of Omicron varied from other VOCs in cell culture, and we needed to develop PRNT_50_ protocols for Omicron compared to other VOCs. Therefore, we will describe our Omicron methodology and present our Omicron PRNT_50_ data in a separate analysis.

Our work provides additional support for using quantitative immunoassays and VLP assays as proxy assays that can replace PRNT_50_ for the purposes of identifying donor plasma with high levels of neutralizing antibodies against SARS-CoV-2 VOCs (3, 7). The study provides additional evidence that plasma approaching 10^4^ BAU/mL also showed strong evidence of functional neutralization of SARS-CoV-2 Delta variants in a culture-based neutralization assay (28). This is not incremental information as similar work done by Harvala et al. used a different immunoassay and a different functional culture-based bioassay (28). Such work is important given the differences in how laboratories have identified high-titer convalescent plasma in the past (5). Taken together, we conclude that the quantitative assay of the Abbott Quant assay and VLP IC_50_ and PRNT_50_ results for wild-type, Alpha, Beta, Gamma, and Delta SARS-CoV-2. An Abbott Quant assay cutoff of 7.1 × 10^3^ BAU/mL or log_3.9_ BAU/mL might detect high-titer plasma against a broad spectrum of wild-type and VOC SARS-CoV-2. Our next steps are to assess how well the Abbott Quant assay quantitatively detects high-titer plasma against Omicron SARS-CoV-2. We have noted several limitations of PRNT_50_ methodologies to identify donor plasma with high levels of anti-SARS-CoV-2 in a timely manner. This has been further elucidated in our prior experiments (3) as well as other studies (28). A combination of VLP assays and quantitative assays that historically have shown correlations to PRNT_50_ results may allow for the development of faster protocols to identify plasma with neutralizing capacity against new VOCs when they arise in the future.

## MATERIALS AND METHODS

### Ethical considerations.

Ethics board clearance for this project was received from the Canadian Blood Services and the University of Alberta and Sinai Health, Toronto (Mount Sinai Hospital).

### CIHR correlates of immunity study participants and samples.

As the blood operator for Canada, except for Quebec, Canadian Blood Services collects blood products in large and small cities. Criteria for blood donors have been described elsewhere (19). Each donation had an additional EDTA-treated plasma (Becton Dickinson, Mississauga, ON, Canada) retention sample collected (7, 35–37).

### Study design and population.

As previously described, this was a repeated cross-sectional design with random cross-sectional sampling of all available retention samples (*n* = 1,500/month) for a 12-month period from January, February, and March of 2021 (total *n* = 4,500) (18). This was followed by sample anonymization. Identified plasma specimens were aliquoted and then transported to test sites and stored at −40 to −80°C for the remainder of the study (37).

### Donor SARS-CoV-2 vaccination history and linking to specific specimens.

At donation, all donors were asked if they had received a SARS-CoV-2 vaccine in the past 3 months as standard practice by Canadian Blood Services. Linkage of vaccine history data to study specimens was undertaken as previously described (19). The source of vaccine (e.g., producer) was not collected, and vaccine data could not be linked to provincial vaccination records.

### Specimens chosen for SARS-CoV-2 neutralization testing.

Specimens assessed for anti-SARS-CoV-2 neutralization capacity were previously selected using a tiered testing approach as previously described (7). Briefly, this approach utilized the Abbott Architect anti-N SARS-CoV-2 IgG assay and the Sinai Health assays (see the supplemental material for further information on assays) (7). This chosen subset of specimens was previously assessed with wild-type and VOC (Alpha, Beta, Gamma, and Delta) SARS-CoV-2 VLP assays. These prior methods utilized are described in the supplemental material (7).

### Definitions of evidence of anti-N positivity.

Serological evidence of anti-N positivity was defined as the presence of an anti-N signal by at least one of the Abbott Architect anti-N SARS-CoV-2 IgG assay or the Sinai Health Anti-N assay (see the supplemental material for further information on assays). These definitions were previously established based on a prior study that assessed the same specimens for wild-type and VOC neutralization using VLP assays (7).

### SARS-CoV-2 antibody testing using the Abbott Quant assay.

Residual plasma samples were then assessed with the Abbott Quant assay (Abbott Laboratories, Chicago, IL, USA) as per the package insert.

### Conversion of Abbott Quant assay results to BAU per milliliter.

Semiquantitative values units per milliliter from the Abbott Quant assay were converted to BAU per milliliter by applying the following conversion factor as previously described (18): Abbott Quant assay (U/mL) × (1/7).

### PRNT_50_ assays: wild type and variants of concern.

Selected EDTA plasma specimens were also analyzed by PRNT_50_ utilizing the Wuhan wild-type (B1, GISAID#EPI_ISL_425177) as well as variant of concern strains (Alpha [B.1.1.7], Beta [B.1.351], Gamma [P.1], and Delta [B.1.617.2]), which was undertaken at the University of Alberta (Edmonton, Canada) as previously described (6). Virus identity was confirmed by whole-genome sequencing. The University of Alberta assay was previously validated to qualify CCP for neutralization of wild-type SARS-CoV-2 in a clinical trial (5). We previously defined high-titer CCP as having a PRNT_50_ of ≥1:160, and low-titer CCP was defined as having a PRNT_50_ of <1:160 (2). In general, the lowest PRNT_50_ results were expressed as <1:20 in the tables within this article. When calculating correlations, PRNT_50_ values of <1:20 were converted to 1:10 to allow for the use of the data.

### Data storage and statistical analysis.

Data storage was undertaken using a Microsoft Excel (Redmond, WA, USA) spreadsheet. GraphPad Prism (9.2.0, GraphPad Software, Inc., San Diego, CA, USA) was used to analyze data.
