# Supplementary material for: Utilization of the Abbott SARS-CoV-2 IgG II Quant Assay To Identify High-Titer Anti-SARS-CoV-2 Neutralizing Plasma against Wild-Type and Variant SARS-CoV-2 Viruses
Source: Microbiol Spectr. 2022 Sep 20;10(5):e02811-22. doi: 10.1128/spectrum.02811-22 (PMC9602363; doi:10.1128/spectrum.02811-22)
Supplement: Supplemental file 1 — Supplemental material. Download spectrum.02811-22-s0001.pdf, PDF file, 1.4 MB [file spectrum.02811-22-s0001.pdf]

## Supplemental materials

**Methods for selection of study specimens.** Specimens had been previously analyzed with Enzyme-linked immunosorbent assays for detecting IgG and virus-like particle assays (VLPs) as described below.

**Enzyme-linked immunosorbent assays for detecting IgG.** As previously described, specimens were analyzed for total IgG antibody levels using spike (anti-S), receptor binding domain (anti-RBD) and nucleocapsid (anti-N) (Lunenfeld-Tanenbaum Research Institute, Sinai Health) (1, 2). Ratio-converted ELISA values were calculated and seropositivity nd cut-offs (positive) or each of the targets were: N (≥0.396), RBD (≥0.186), S (≥0.190) (3). Plasma samples were also tested with the Abbott Architect SARS-CoV-2 IgG test (Abbott Laboratories, USA) which detects anti-N IgG antibodies. Testing was undertaken as per manufacturers instructions using an antibody index (AI) cut-off of 1.4 (3, 4). A specimen was defined as anti-N positive if it had one or more anti-N positive signals from either the Abbott or the Lunenfeld-Tanenbaum Research Institute, Sinai Health assays.

**Selection of specimens for neutralizing analysis.** The tiered testing approach selected specimens with any potential evidence of an anti-S or anti-RBD signal (with or without anti-N) (5, 6). Randomization of specimens was then undertaken with replacement (1, 3).

**Spike-pseudotyped VLP neutralization assay.** Pseudovirus generation in HEK293TN cells and the pseudovirus neutralization assay were previously described (7). These methods were used by our group with minor modifications as previously described (1). Spike protein constructs including Wildtype Wuhan-1 and VOCs: Alpha, B.1.1.7; Beta, B.1.351; Gamma, P.1 and Delta, B.1.617.2 were utilized as previously described (1). These were kindly provided by W. Rod Hardy of CoVaRR-Net.

**Results from the prior study (provided for context).**

From 4500 specimens, our approach determined that 320 (7.1%) specimens were positive for anti-S and/or anti-RBD IgG (3). In the group with a history of vaccine, 111/112 (99.1%) specimens were anti-S positive; 104 specimens were also anti-RBD positive. In this group, 1/112 specimens was anti-S negative and anti-RBD positive. In the unvaccinated group, a total of 254/278 (91.4%) specimens were anti-S positive; 123 were anti-RBD positive. In the unvaccinated group, 24/278 specimens were anti-RBD positive and anti-S negative (1).

**Supplemental results**

**Results**

**TABLE S1** Summary of previous immunoassay results (1) in a subset of specimens chosen and available for testing with the Abbott Quant assay and $PRNT_{50}$ (January-March 2021).

| Sample ID | Vaccinated | Anti-S ratio | Anti-S | Anti-RBD ratio | Anti-RBD | | N (Sinai Health) ratio | Anti-N (Sinai Health) | Abbott anti-N ratio | Abbott anti-N |
|---|---|---|---|---|---|---|---|---|---|---|
| CIHR013654 | Yes | 1.69 | Pos | 1.41 | Pos | | 0.41 | Pos | 0.02 | Neg |
| CIHR013818 | Yes | 1.08 | Pos | 0.77 | Pos | | 0.03 | Neg | 0.01 | Neg |
| CIHR014329 | Yes | 1.86 | Pos | 1.88 | Pos | | 0.03 | Neg | 0.02 | Neg |
| CIHR015234 | Yes | 1.48 | Pos | 0.75 | Pos | | 0.09 | Neg | 0.01 | Neg |
| CIHR015533 | Yes | 1.64 | Pos | 1.81 | Pos | | 0.04 | Neg | 0.09 | Neg |
| CIHR015657 | Yes | 1.64 | Pos | 1.60 | Pos | | 0.06 | Neg | 0.09 | Neg |
| CIHR015884 | Yes | 1.88 | Pos | 1.90 | Pos | | 0.34 | Neg | 0.13 | Neg |
| CIHR015946 | Yes | 1.24 | Pos | 0.61 | Pos | | 0.60 | Pos | 0.03 | Neg |
| CIHR015958 | Yes | 1.77 | Pos | 1.57 | Pos | | 0.02 | Neg | 0.03 | Neg |
| CIHR016698 | Yes | 1.74 | Pos | 1.76 | Pos | | 0.01 | Neg | 0.04 | Neg |
| CIHR016894 | Yes | 1.49 | Pos | 1.59 | Pos | | 1.60 | Pos | 5.52 | Pos |
| CIHR016904 | Yes | 1.13 | Pos | 0.13 | Neg | | 0.02 | Neg | 0.01 | Neg |
| CIHR016905 | Yes | 1.48 | Pos | 1.51 | Pos | | 0.01 | Neg | 0.04 | Neg |
| CIHR016930 | Yes | 0.04 | Neg | 0.25 | Pos | | 0.05 | Neg | 0.01 | Neg |
| CIHR017087 | Yes | 1.61 | Pos | 1.49 | Pos | | 0.01 | Neg | 0.03 | Neg |
| CIHR017189 | Yes | 1.58 | Pos | 1.41 | Pos | | 0.05 | Neg | 0.03 | Neg |
| CIHR017229 | Yes | 0.31 | Pos | 0.09 | Neg | | 0.03 | Neg | 0.01 | Neg |
| CIHR017333 | Yes | 1.69 | Pos | 1.71 | Pos | | 0.40 | Pos | 0.03 | Neg |
| CIHR017534 | Yes | 0.93 | Pos | 0.31 | Pos | | 0.03 | Neg | 0.03 | Neg |
| CIHR017540 | Yes | 1.53 | Pos | 1.37 | Pos | | 0.05 | Neg | 0.03 | Neg |
| CIHR017728 | Yes | 1.82 | Pos | 1.83 | Pos | | 0.11 | Neg | 0.04 | Neg |
| CIHR017730 | Yes | 1.65 | Pos | 1.73 | Pos | | 1.94 | Pos | 5.31 | Pos |
| CIHR017824 | Yes | 1.50 | Pos | 1.61 | Pos | | 0.02 | Neg | 0.03 | Neg |

| | | | | | | | | | | |
|---|---|---|---|---|---|---|---|---|---|---|
| CIHR017838 | Yes | 1.53 | Pos | 1.53 | Pos | | 0.02 | Neg | 0.05 | Neg |
| CIHR018126 | Yes | 1.70 | Pos | 1.50 | Pos | | 0.07 | Neg | 0.06 | Neg |
| CIHR013757 | No | 0.67 | Pos | 0.33 | Pos | | 0.53 | Pos | 1.13 | Neg |
| CIHR013936 | No | 1.43 | Pos | 0.67 | Pos | | 0.43 | Pos | 1.32 | Neg |
| CIHR014110 | No | 0.68 | Pos | 0.15 | Neg | | 1.60 | Pos | 2.74 | Pos |
| CIHR014113 | No | 1.49 | Pos | 0.84 | Pos | | 1.40 | Pos | 3.08 | Pos |
| CIHR014235 | No | 1.44 | Pos | 1.14 | Pos | | 1.01 | Pos | 2.76 | Pos |
| CIHR014238 | No | 0.28 | Pos | 0.04 | Neg | | 0.02 | Neg | 0.03 | Neg |
| CIHR014309 | No | 1.75 | Pos | 1.66 | Pos | | 1.86 | Pos | 4.59 | Pos |
| CIHR014491 | No | 0.25 | Pos | 0.06 | Neg | | 0.05 | Neg | 0.02 | Neg |
| CIHR014632 | No | 0.42 | Pos | 0.10 | Neg | | 0.10 | Neg | 0.3 | Neg |
| CIHR014664 | No | 0.20 | Pos | 0.02 | Neg | | 0.09 | Neg | 0.03 | Neg |
| CIHR014834 | No | 1.39 | Pos | 1.23 | Pos | | 2.03 | Pos | 6.45 | Pos |
| CIHR014840 | No | 1.21 | Pos | 1.82 | Pos | | 0.87 | Pos | 2.32 | Pos |
| CIHR014884 | No | 1.21 | Pos | 0.84 | Pos | | 0.52 | Pos | 2.56 | Pos |
| CIHR014926 | No | 0.52 | Pos | 0.04 | Neg | | 0.35 | Neg | 0.01 | Neg |
| CIHR014993 | No | 0.62 | Pos | 0.17 | Neg | | 1.84 | Pos | 0.97 | Neg |
| CIHR015079 | No | 0.19 | Pos | 0.07 | Neg | | 0.17 | Neg | 0.06 | Neg |
| CIHR015094 | No | 1.34 | Pos | 1.11 | Pos | | 1.34 | Pos | 5.17 | Pos |
| CIHR015434 | No | 0.36 | Pos | 0.36 | Pos | | 1.17 | Pos | 3.09 | Pos |
| CIHR015475 | No | 0.27 | Pos | 0.03 | Neg | | 0.09 | Neg | 0.01 | Neg |
| CIHR015843 | No | 0.31 | Pos | 0.02 | Neg | | 0.02 | Neg | 0.01 | Neg |
| CIHR015948 | No | 0.30 | Pos | 0.02 | Neg | | 0.05 | Neg | 0.17 | Neg |
| CIHR016024 | No | 1.05 | Pos | 0.42 | Pos | | 0.50 | Pos | 2.23 | Pos |
| CIHR016403 | No | 0.22 | Pos | 0.06 | Neg | | 0.13 | Neg | 0.02 | Neg |
| CIHR016447 | No | 0.18 | No | 0.19 | Pos | | 0.30 | Neg | 0.06 | Neg |
| CIHR016548 | No | 0.47 | Pos | 0.11 | Neg | | 0.16 | Neg | 0.05 | Neg |
| CIHR016557 | No | 0.22 | Pos | 0.02 | Neg | | 0.04 | Neg | 0.03 | Neg |
| CIHR016624 | No | 1.17 | Pos | 0.56 | Pos | | 1.26 | Pos | 2.82 | Pos |
| CIHR016973 | No | 0.27 | Pos | 0.01 | Neg | | 0.07 | Neg | 0.3 | Neg |
| CIHR016979 | No | 0.79 | Pos | 0.24 | Pos | | 0.53 | Pos | 1.66 | Pos |

| | | | | | | | | | | |
|---|---|---|---|---|---|---|---|---|---|---|
| CIHR017127 | No | 0.33 | Pos | 0.40 | Pos | | 0.55 | Pos | 0.06 | Neg |
| CIHR017305 | No | 1.11 | Pos | 0.67 | Pos | | 2.09 | Pos | 6.95 | Pos |
| CIHR017530 | No | 0.46 | Pos | 0.01 | Neg | | 0.04 | Neg | 0.1 | Neg |
| CIHR017724 | No | 0.97 | Pos | 0.51 | Pos | | 0.82 | Pos | 1.96 | Pos |
| CIHR017894 | No | 1.55 | Pos | 1.14 | Pos | | 0.52 | Pos | 3.1 | Pos |
| CIHR017945 | No | 0.19 | Pos | 0.01 | Neg | | 0.01 | Neg | 0.02 | Neg |
| CIHR017990 | No | 1.00 | Pos | 0.37 | Pos | | 0.97 | Pos | 2.61 | Pos |
| CIHR018000 | No | 0.26 | Pos | 0.12 | Neg | | 0.31 | Neg | 0.88 | Neg |
| CIHR018166 | No | 0.25 | Pos | 0.01 | Neg | | 0.02 | Neg | 0.01 | Neg |
| CIHR018178 | No | 0.22 | Pos | 0.01 | Neg | | 0.02 | Neg | 0.03 | Neg |

**TABLE S2** Summary of Wildtype and VOC VLP $ID_{50}$ results (1) in vaccinated and unvaccinated blood donors (January-March 2021).

| Specimen # | Vaccinated | Anti-N | $ID_{50}$ | | | | |
|---|---|---|---|---|---|---|---|
| | | | Wildtype | Alpha | Beta | Gamma | Delta |
| Vaccinated + any of (RBD +/- S) any N | | | | | | | |
| CIHR013654 | Yes | Yes | $1.51 \times 10^2$ | $3.36 \times 10^1$ | 1.00 | 1.00 | $1.08 \times 10^1$ |
| CIHR015946 | Yes | Yes | $3.96 \times 10^1$ | 1.00 | 1.00 | 1.00 | 1.00 |
| CIHR016894 | Yes | Yes | $5.75 \times 10^4$ | $2.55 \times 10^4$ | $1.20 \times 10^4$ | $2.65 \times 10^4$ | $3.02 \times 10^4$ |
| CIHR017333 | Yes | Yes | $4.45 \times 10^3$ | $2.37 \times 10^3$ | $2.81 \times 10^2$ | $1.70 \times 10^3$ | $2.97 \times 10^3$ |
| CIHR017730 | Yes | Yes | $1.62 \times 10^4$ | $7.26 \times 10^3$ | $4.00 \times 10^3$ | $5.35 \times 10^3$ | $6.24 \times 10^3$ |
| Vaccinated + any of (RBD +/- S) no N | | | | | | | |
| CIHR013818 | Yes | No | $2.13 \times 10^3$ | $1.54 \times 10^3$ | 1.00 | 1.00 | $2.65 \times 10^3$ |
| CIHR014329 | Yes | No | $2.45 \times 10^2$ | $1.12 \times 10^2$ | $2.74 \times 10^1$ | $9.17 \times 10^1$ | $2.22 \times 10^2$ |
| CIHR015234 | Yes | No | $1.07 \times 10^2$ | 1.00 | 1.00 | 1.00 | 1.00 |
| CIHR015533 | Yes | No | $1.83 \times 10^4$ | $6.41 \times 10^3$ | $6.81 \times 10^2$ | $3.17 \times 10^3$ | $5.14 \times 10^3$ |
| CIHR015657 | Yes | No | $3.97 \times 10^2$ | $1.11 \times 10^2$ | $3.89 \times 10^1$ | $1.24 \times 10^2$ | $1.51 \times 10^2$ |

| | | | | | | | |
|---|---|---|---|---|---|---|---|
| CIHR015884 | Yes | No | $1.98 \times 10^3$ | $7.16 \times 10^2$ | $2.07 \times 10^2$ | $9.97 \times 10^2$ | $1.28 \times 10^3$ |
| CIHR015958 | Yes | No | $1.12 \times 10^3$ | $1.85 \times 10^2$ | 1.00 | $7.90 \times 10^1$ | $1.90 \times 10^2$ |
| CIHR016698 | Yes | No | $1.48 \times 10^3$ | $1.01 \times 10^3$ | $1.23 \times 10^2$ | $7.84 \times 10^2$ | $7.07 \times 10^2$ |
| CIHR016904 | Yes | No | $8.13 \times 10^1$ | $2.17 \times 10^1$ | 1.00 | 1.00 | $5.39 \times 10^1$ |
| CIHR016905 | Yes | No | $4.70 \times 10^3$ | $2.42 \times 10^3$ | $4.99 \times 10^2$ | $9.92 \times 10^2$ | $2.39 \times 10^3$ |
| CIHR016930 | Yes | No | 1.00 | 1.00 | 1.00 | 1.00 | 1.00 |
| CIHR017087 | Yes | No | $3.59 \times 10^2$ | $1.20 \times 10^2$ | $7.70 \times 10^1$ | $3.14 \times 10^2$ | $4.06 \times 10^2$ |
| CIHR017189 | Yes | No | $1.18 \times 10^3$ | $4.24 \times 10^2$ | $9.15 \times 10^1$ | $6.30 \times 10^2$ | $5.13 \times 10^2$ |
| CIHR017229 | Yes | No | 1.00 | 1.00 | 1.00 | 1.00 | 1.00 |
| CIHR017534 | Yes | No | $5.53 \times 10^1$ | $4.89 \times 10^1$ | 1.00 | 1.00 | 1.00 |
| CIHR017540 | Yes | No | $6.69 \times 10^2$ | $1.41 \times 10^2$ | 1.00 | $1.02 \times 10^2$ | $7.45 \times 10^1$ |
| CIHR017728 | Yes | No | $1.13 \times 10^4$ | $4.73 \times 10^3$ | $8.37 \times 10^2$ | 2.37 x 103 | $2.67 \times 10^3$ |
| CIHR017824 | Yes | No | $1.40 \times 10^3$ | $4.30 \times 10^2$ | $2.65 \times 10^2$ | $6.54 \times 10^2$ | $3.09 \times 10^2$ |
| CIHR017838 | Yes | No | $9.35 \times 10^3$ | $2.79 \times 10^3$ | $6.21 \times 10^2$ | $1.82 \times 10^3$ | $2.02 \times 10^3$ |
| CIHR018126 | Yes | No | $7.97 \times 10^2$ | $5.62 \times 10^2$ | $1.15 \times 10^2$ | $5.17 \times 10^2$ | $3.09 \times 10^2$ |
| Unvaccinated + any of (RBD +/- S) any N | | | | | | | |

| | | | | | | | |
|---|---|---|---|---|---|---|---|
| CIHR013757 | No | Yes | 1.00 | 1.00 | 1.00 | 1.00 | 1.00 |
| CIHR013936 | No | Yes | $4.91 \times 10^{2}$ | $1.93 \times 10^{2}$ | $8.09 \times 10^{1}$ | $1.44 \times 10^{2}$ | $1.55 \times 10^{2}$ |
| CIHR014110 | No | Yes | $7.02 \times 10^{2}$ | $1.25 \times 10^{2}$ | $2.29 \times 10^{2}$ | $3.94 \times 10^{2}$ | $1.24 \times 10^{2}$ |
| CIHR014113 | No | Yes | $1.27 \times 10^{3}$ | $1.39 \times 10^{2}$ | $5.06 \times 10^{1}$ | $3.03 \times 10^{2}$ | $1.41 \times 10^{2}$ |
| CIHR014235 | No | Yes | $1.01 \times 10^{3}$ | $3.76 \times 10^{2}$ | $3.74 \times 10^{1}$ | $6.23 \times 10^{1}$ | $9.52 \times 10^{2}$ |
| CIHR014309 | No | Yes | $3.39 \times 10^{2}$ | $1.28 \times 10^{2}$ | $5.06 \times 10^{1}$ | $1.88 \times 10^{2}$ | $1.3 \times 10^{2}$ |
| CIHR014840 | No | Yes | $1.85 \times 10^{2}$ | $9.53 \times 10^{1}$ | $2.33 \times 10^{1}$ | $4.38 \times 10^{1}$ | $5.43 \times 10^{1}$ |
| CIHR014884 | No | Yes | $5.65 \times 10^{2}$ | $1.21 \times 10^{2}$ | $3.12 \times 10^{1}$ | $3.29 \times 10^{2}$ | $1.22 \times 10^{2}$ |
| CIHR014993 | No | Yes | 1.00 | 1.00 | 1.00 | 1.00 | 1.00 |
| CIHR015094 | No | Yes | $2.08 \times 10^{3}$ | $1.12 \times 10^{3}$ | $3.62 \times 10^{3}$ | $1.47 \times 10^{3}$ | $4.42 \times 10^{3}$ |
| CIHR015434 | No | Yes | $2.71 \times 10^{2257}$ | $1.10 \times 10^{2}$ | $5.04 \times 10^{1}$ | $1.52 \times 10^{2}$ | $1.42 \times 10^{2}$ |
| CIHR016024 | No | Yes | $2.69 \times 10^{2}$ | $3.41 \times 10^{2}$ | 1.00 | $3.60 \times 10^{1}$ | $4.24 \times 10^{2}$ |
| CIHR016624 | No | Yes | $7.33 \times 10^{1}$ | $5.24 \times 10^{1}$ | $2.57 \times 10^{1}$ | $4.62 \times 10^{1}$ | $8.38 \times 10^{1}$ |
| CIHR016979 | No | Yes | $2.80 \times 10^{2}$ | $2.25 \times 10^{2}$ | 1.00 | $4.60 \times 10^{2}$ | $1.12 \times 10^{3}$ |
| CIHR017127 | No | Yes | $7.31 \times 10^{1}$ | $3.50 \times 10^{1}$ | 1.00 | 7.84 | 1.00 |
| CIHR017305 | No | Yes | $2.57 \times 10^{2}$ | $1.41 \times 10^{2}$ | $3.73 \times 10^{1}$ | $1.47 \times 10^{2}$ | $1.44 \times 10^{2}$ |
| CIHR017724 | No | Yes | $2.57 \times 10^{3}$ | $1.26 \times 10^{3}$ | $3.68 \times 10^{2}$ | $7.59 \times 10^{2}$ | $5.56 \times 10^{2}$ |
| CIHR017894 | No | Yes | $8.78 \times 10^{1}$ | $8.06 \times 10^{1}$ | $3.92 \times 10^{1}$ | $5.59 \times 10^{1}$ | $4.22 \times 10^{1}$ |

| | | | | | | | |
|---|---|---|---|---|---|---|---|
| CIHR017990 | No | Yes | $3.74 \times 10^{2}$ | $1.46 \times 10^{2}$ | $4.10 \times 10^{1}$ | $1.17 \times 10^{2}$ | $6.43 \times 10^{1}$ |
| Unvaccinated + any of (RBD +/- S) no N | | | | | | | |
| CIHR014238 | No | No | 1.00 | 1.00 | 1.00 | 1.00 | 1.00 |
| CIHR014491 | No | No | 1.00 | 1.00 | 1.00 | 1.00 | 1.00 |
| CIHR014632 | No | No | 1.00 | 1.00 | 1.00 | 1.00 | 1.00 |
| CIHR014664 | No | No | 1.00 | 1.00 | 1.00 | 1.00 | 1.00 |
| CIHR014926 | No | No | 1.00 | 1.00 | 1.00 | 1.00 | 1.00 |
| CIHR015079 | No | No | 1.00 | 1.00 | 1.00 | 1.00 | 1.00 |
| CIHR015475 | No | No | 1.00 | 1.00 | 1.00 | 1.00 | 1.00 |
| CIHR015843 | No | No | 1.00 | 1.00 | 1.00 | 1.00 | 1.00 |
| CIHR015948 | No | No | 1.00 | 1.00 | 1.00 | 1.00 | 1.00 |
| CIHR016403 | No | No | 1.00 | 1.00 | 1.00 | 1.00 | 1.00 |
| CIHR016447 | No | No | 1.00 | 1.00 | 1.00 | 1.00 | 1.00 |
| CIHR016548 | No | No | 1.00 | 1.00 | 1.00 | 1.00 | 1.00 |
| CIHR016557 | No | No | 1.00 | 1.00 | 1.00 | 1.00 | 1.00 |
| CIHR016973 | No | No | 1.00 | 1.00 | 1.00 | 1.00 | 1.00 |
| CIHR017530 | No | No | 1.00 | 1.00 | 1.00 | 1.00 | 1.00 |

| | | | | | | | |
|---|---|---|---|---|---|---|---|
| CIHR017945 | No | No | 1.00 | 1.00 | 1.00 | 1.00 | 1.00 |
| CIHR018000 | No | No | 1.00 | 1.00 | 1.00 | 1.00 | 1.00 |
| CIHR018166 | No | No | 1.00 | 1.00 | 1.00 | 1.00 | 1.00 |
| CIHR018178 | No | No | 1.00 | 1.00 | 1.00 | 1.00 | 1.00 |

**TABLE S3** Summary of Abbott Quant assay and $PRNT_{50}$ results: Vaccinated + anti-N.

| Specimen # | Vaccination history | $PRNT_{50}$ against variants | | | | | Abbott Quant assay |
|---|---|---|---|---|---|---|---|
| | | Wuhan | Alpha | Beta | Gamma | Delta | BAU/ml |
| CIHR013654 | 1 dose ≥ 14 days | 40 | <20 | <20 | <20 | <20 | 181.9 |
| CIHR015946 | Dose and timing NA | 20 | <20 | <20 | <20 | <20 | 70.7 |
| CIHR016894 | Dose and timing NA | 5120 | 2560 | 5120 | 5120 | 5120 | 7142.9 |
| CIHR017333 | Dose and timing NA | 1280 | 640 | 160 | 160 | 160 | 2783.4 |
| CIHR017730 | Dose and timing NA | 2560 | 1280 | 1280 | 2560 | 1280 | 3789.6 |

NA, not available

**TABLE S4** Summary of Abbott Quant assay and $PRNT_{50}$ results: Vaccinated + No anti-N.

| Specimen # | Vaccination history | $PRNT_{50}$ against variants | | | | | Abbott Quant assay |
|---|---|---|---|---|---|---|---|
| | | Wuhan | Alpha | Beta | Gamma | Delta | BAU/ml |
| CIHR013818 | Dose and timing NA | 40 | 80 | <20 | <20 | 80 | 189.3 |
| CIHR014329 | Dose and timing NA | 40 | 20 | 40 | <20 | 40 | 256.4 |
| CIHR015234 | 1 dose (≥14 days) | <20 | <20 | <20 | <20 | <20 | 118.8 |
| CIHR015533 | 1 dose (≥14 days) | 640 | 640 | 320 | 160 | 640 | 7142.9 |
| CIHR015657 | Dose and timing NA | 40 | 20 | 20 | <20 | 20 | 761.6 |
| CIHR015884 | Fully vaccinated | 320 | 320 | 160 | 160 | 160 | 2432.0 |
| CIHR015958 | Dose and timing NA | 40 | 40 | 20 | <20 | 20 | 523.4 |
| CIHR016698 | 1 dose (>=14 days) | 320 | 320 | 80 | 160 | 80 | 1886.0 |
| CIHR016904 | Dose and timing NA | 20 | 20 | <20 | <20 | 20 | 19.3 |
| CIHR016905 | Fully vaccinated | 320 | 640 | 160 | 160 | 160 | 3392.0 |
| CIHR016930 | 1 dose (≥14 days) | <20 | <20 | <20 | <20 | 20 | 0.0 |
| CIHR017087 | Dose and timing NA | 80 | 80 | 80 | 20 | 80 | 468.5 |
| CIHR017189 | 1 dose (≥14 days) | 160 | 80 | 80 | 80 | 80 | 1167.7 |
| CIHR017229 | Dose and timing NA | <20 | <20 | 20 | <20 | <20 | 11.3 |
| CIHR017534 | Dose and timing NA | <20 | <20 | <20 | <20 | <20 | 45.7 |
| CIHR017540 | Dose and timing NA | 80 | 40 | 20 | <20 | <20 | 447.6 |
| CIHR017728 | Dose and timing NA | 1280 | 640 | 320 | 160 | 640 | 3936.3 |
| CIHR017824 | Dose and timing NA | 160 | 160 | 160 | 80 | 80 | 728.7 |
| CIHR017838 | Dose and timing NA | 640 | 640 | 640 | 320 | 640 | 2741.1 |
| CIHR018126 | Dose and timing NA | 80 | 80 | 80 | 20 | 40 | 562.8 |

NA, not available

**TABLE S5** Summary of Abbott Quant assay and $PRNT_{50}$ results: Not Vaccinated + anti-N.

| Specimen # | $PRNT_{50}$ against variants | | | | | Abbott Quant assay |
|---|---|---|---|---|---|---|
| | Wuhan | Alpha | Beta | Gamma | Delta | BAU/ml |
| CIHR013757 | <20 | <20 | <20 | <20 | <20 | 2.3 |
| CIHR013936 | 80 | 40 | 40 | 40 | 40 | 66.7 |
| CIHR014110 | 80 | 20 | 40 | 40 | 40 | 176.9 |
| CIHR014113 | 80 | 20 | 20 | 40 | 40 | 227.5 |
| CIHR014235 | 160 | 40 | <20 | <20 | 320 | 301.8 |
| CIHR014309 | 80 | 40 | 20 | <20 | 80 | 294.2 |
| CIHR014840 | 40 | <20 | <20 | <20 | 20 | 105.7 |
| CIHR014884 | 160 | 160 | <20 | 80 | 80 | 182.5 |
| CIHR014993 | <20 | <20 | <20 | <20 | <20 | 13.4 |
| CIHR015094 | 320 | 320 | 640 | 80 | 640 | 46.7 |
| CIHR015434 | 40 | 40 | <20 | <20 | 40 | 59.0 |
| CIHR016024 | 20 | 20 | <20 | <20 | <20 | 69.8 |
| CIHR016624 | 20 | 20 | 40 | 20 | 20 | 23.6 |
| CIHR016979 | 40 | 40 | 20 | 80 | 80 | 113.6 |
| CIHR017127 | <20 | 20 | <20 | <20 | 40 | 22.2 |
| CIHR017305 | 80 | 40 | 20 | <20 | 80 | 106.8 |
| CIHR017724 | 160 | 80 | 80 | 80 | 160 | 285.7 |
| CIHR017894 | 40 | 20 | <20 | <20 | 20 | 47.5 |
| CIHR017990 | 80 | 40 | 20 | 20 | 40 | 81.9 |

**TABLE S6** Summary of Abbott Quant assay and $PRNT_{50}$ results: Not vaccinated + No anti-N.

| Specimen # | $PRNT_{50}$ against variants | | | | | Abbott Quant assay |
|---|---|---|---|---|---|---|
| | Wuhan | Alpha | Beta | Gamma | Delta | BAU/ml |
| CIHR014238 | <20 | <20 | <20 | <20 | <20 | 0.0 |
| CIHR014491 | <20 | <20 | <20 | <20 | <20 | 0.0 |
| CIHR014632 | <20 | <20 | <20 | <20 | <20 | 10.4 |
| CIHR014664 | <20 | <20 | <20 | <20 | <20 | 0.3 |
| CIHR014926 | <20 | <20 | <20 | <20 | <20 | 0.2 |
| CIHR015079 | <20 | <20 | <20 | <20 | <20 | 0.2 |
| CIHR015475 | <20 | <20 | <20 | <20 | <20 | 0.3 |
| CIHR015843 | <20 | <20 | <20 | <20 | <20 | 0.0 |
| CIHR015948 | <20 | <20 | <20 | <20 | <20 | 0.2 |
| CIHR016403 | <20 | <20 | <20 | <20 | <20 | 0.2 |
| CIHR016447 | <20 | <20 | <20 | <20 | <20 | 0.0 |
| CIHR016548 | <20 | <20 | <20 | <20 | <20 | 1.6 |
| CIHR016557 | <20 | <20 | <20 | <20 | <20 | 0.5 |
| CIHR016973 | <20 | <20 | <20 | <20 | <20 | 0.0 |
| CIHR017530 | <20 | <20 | <20 | <20 | <20 | 0.0 |
| CIHR017945 | <20 | <20 | <20 | <20 | <20 | 0.1 |
| CIHR018000 | <20 | <20 | <20 | <20 | <20 | 14.2 |
| CIHR018166 | <20 | <20 | <20 | <20 | <20 | 1.2 |
| CIHR018178 | <20 | <20 | <20 | <20 | <20 | 0.0 |

**Supplement References**

1. Drews SJ, Hu Q, Samson R, Abe KT, Rathod B, Colwill K, et al. SARS-CoV-2 virus-like particle neutralizing capacity in blood donors depends on serological profile and donor-declared SARS-CoV-2 vaccination history. Microbiol Spectr. 2022;10(1):e0226221.

2. Colwill K, Galipeau Y, Stuible M, Gervais C, Arnold C, Rathod B, et al. A scalable serology solution for profiling humoral immune responses to SARS-CoV-2 infection and vaccination. Clin Transl Immunology. 2022;11(3):e1380.

3. Saeed S, O'Brien SF, Abe K, Yi QL, Rathod B, Wang J, et al. Severe acute respiratory syndrome coronavirus 2 (SARS-CoV-2) seroprevalence: Navigating the absence of a gold standard. PLoS One. 2021;16(9):e0257743.

4. Saeed S, Drews S, Pambrun C, Yi Q, Osmond L, O'Brien S. SARS-CoV-2 seroprevalence among blood donors after the first COVID-19 wave in Canada. Transfusion. 2021;61(3):862-72.

5. Abe K, Li Z, Samson R, Samavarchi-Tehrani P, Valcourt E, Wood H, et al. A simple protein-based surrogate neutralization assay for SARS-CoV-2. JCI Insight. 2020;5(19).

6. Isho B, Abe K, Zuo M, Jamal A, Rathod B, Wang J, et al. Persistence of serum and saliva antibody responses to SARS-CoV-2 spike antigens in COVID-19 patients. Sci Immunol. 2020;5(52).

7. Abe K, Hu Q, Mozafarihashjin M, Samson R, Manguiat K, Robinson A, et al. Neutralizing antibody responses to SARS-CoV-2 variants in vaccinated Ontario long-term care home residents and workers. medRxiv. 2021:2021.08.06.21261721.

**doi:** https://doi.org/10.1101/2021.08.06.21261721
